# An Early Lexical Screening Tool for British English: Psychometric Properties and Clinical Utility

**DOI:** 10.1111/1460-6984.70287

**Published:** 2026-07-07

**Authors:** Allegra Cattani, Dora Bianchi, Caroline Floccia

**Affiliations:** ^1^ Department of Developmental and Social Psychology Sapienza University of Rome Rome Italy; ^2^ School of Psychology University of Plymouth Plymouth UK

**Keywords:** early screening, gender differences, lexicon, psychometric properties, sensitivity and specificity, toddlers

## Abstract

**Background:**

Existing direct measures of vocabulary for ages 2–3 years are insufficient as vocabulary increases to include nouns, verbs, adjectives and adverbs, reflecting qualitative changes that support emerging grammatical abilities.

**Aims:**

The present study aims to establish the psychometric properties and validate a lexical screening tool for 19–36‐month‐old British English‐speaking children, the WinG (Words in Game) test. Specifically, it examines gender differences in internal consistency, concurrent validity and predictive gender invariance across comprehension and production tasks. In addition, the study evaluates the diagnostic accuracy of the tool by establishing clinical cut‐off scores to distinguish children with typical versus at‐risk language development. Finally, it presents developmental trends in toddlers’ aggregate lexical comprehension and production scores, reported in percentiles and stratified by gender.

**Methods and Procedures:**

Participants were 336 English‐speaking children aged 19–36 months. Sub‐set of 85 children were screened with the Words in Game (WinG) test and the Preschool Language Scale (PLS‐4). The PLS‐4 scores were used as reference tests to ascertain psychometric properties and estimate the sensitivity and specificity of the WinG comprehension and production tasks.

**Outcomes and Results:**

Girls outperformed boys on both comprehension and production tasks. The WinG comprehension and production tasks showed strong concurrent validity, with scores positively and highly correlated with the PLS‐4 reference measures. Predictive gender invariance was also demonstrated, as both WinG dimensions predicted PLS‐4 scores equivalently across genders. Using the 15th percentile as a cut‐off, the WinG test demonstrated fair sensitivity for comprehension and good sensitivity for production, though specificity was below the desirable threshold for both tasks.

**Conclusions and Implications:**

The tool provides a valuable starting point for identifying early receptive and expressive lexical difficulties through direct assessment by speech and language therapists (SLTs). Clinical implications and limitations are also discussed.

**WHAT THIS PAPER ADDS:**

*What is already known on this subject*

- Lexical receptive and expressive abilities emerge and expand rapidly in the second and third years of life. Clinicians and Speech and Language therapists (SLTs) need a systematic, reliable tool to observe the early lexical skills of toddlers.

*What this study adds to existing knowledge*

- This study established the psychometric properties and validated the Words in Game (WinG) test from a large sample of 19–36‐month‐old British English learning children to measure the receptive and expressive nouns and predicates (verbs, adjectives and adverbs). The PLS‐4 scores were used as a reference standard test.
- The WinG comprehension and production tasks showed strong concurrent validity, with scores positively and highly correlated with the PLS‐4 reference measures. The developmental trends in toddlers’ lexical comprehension and production scores are reported in percentiles and stratified by gender. The diagnostic accuracy of the WinG test was ascertained, performing receiver operating curves (ROC) on comprehension and production WinG tasks, entering the 15th percentile clinical cut‐off scores, as indicated by the PLS‐4 reference measures.

*What are the clinical implications of this study?*

- The WinG test is a valid tool designed to screen the comprehension and production of words of British English children in the second and third years of life. The WinG test is a tool to be administered by professionals with expertise in language development, such as SLTs. Thanks to its systematic, direct observation approach and validation against established measures, the WinG test demonstrates fair psychometric properties. It provides SLTs with a reliable tool for early screening of toddlers’ lexical skills to identify language delays or disorders.

## 1 Introduction

Language delays or disorders in the early phases of language development are the most frequent developmental problems in toddlers, with a prevalence varying between 5% and 12% (Law et al. 2000; Mattson et al. 2001). At age 4, Norbury et al. (2016) found that language disorders affect approximately 7.6% of the children's population. Children who are slow to develop expressive language may need further assessment to determine the nature and the severity of language disorders, to provide subsequent support and intervention. Persistent language delays or disorders negatively affect other areas of behaviour and socio‐emotional and academic aspects of children's lives (Beitchman et al. 1996; Brownlie et al. 2016).

Given the emergence and rapid expansion of lexical receptive and expressive abilities in the second and third years of life, the size of the lexicon is a sensitive marker of early language development at age 2 (Patrucco‐Nanchen et al. 2019; Vehkavuori and Stolt 2019). Early vocabulary size is found to be a robust predictor of later language skills (e.g., Conboy and Thal 2006; Marchman and Fernald 2008) and subsequent language impairment (e.g., Dale et al. 2003), making it a reasonable proxy of language development skills in toddlers (see also Cattani et al. 2014).

Language development is understood as an emergent process shaped by children's active engagement with their physical and social environments (Bates et al. 1994; Rowland et al. 2026). As children accumulate linguistic and experiential input, their vocabulary increasingly includes predicate terms (verbs, adjectives and adverbs) relative to nouns, reflecting more complex event representations and semantic networks linked to grammatical organisation. In addition, comprehension and production of words are related but partially dissociable dimensions of lexical knowledge (Bates et al. 1994). Comprehension reflects the consolidation of form‐meaning mappings and conceptual representations, whereas production requires additional processes of lexical retrieval, phonological encoding and articulatory planning. Screening both provides a comprehensive knowledge of the child's lexical system. The current study aims to establish the psychometric properties and validate a lexical screening tool for 19‐ to 36‐month‐old British English‐speaking children, while also examining gender differences. The tool assesses word comprehension and production (including nouns, verbs, adjectives and adverbs) as an initial step towards guiding early interventions and evaluating school readiness by age 4 for the diagnosis of Developmental Language Disorder (DLD).

### 1.1 Early Language Screening Tools

Direct measures of vocabulary for children aged 2–3 years are practically non‐existent (Law and Roy 2008). As vocabulary increases at this age, the repertoire—initially dominated by nouns—expands to include more predicates (verbs, adjectives and adverbs), reflecting qualitative changes that support emerging grammatical abilities (Marchman and Thal 2005).

In Italian, a picture naming test has been normed on a sample of typically developing children (PinG test, Bello et al. 2012) and used as a clinical tool to screen the lexical comprehension and production of children aged 19–36 months. The PinG test comprises four subtests: noun comprehension, predicate comprehension, noun production and predicate production. It has been used to explore the construction of semantic knowledge in developmentally at‐risk preterm children (Sansavini et al. 2015) and children with Down syndrome (Bello et al. 2014; Stefanini et al. 2007), as well as to examine the semantic construction underlying lexical naming in cross‐cultural studies of co‐speech gestures (Cattani, Floccia et al. 2019; Marentette et al. 2016; Pettenati et al. 2012).

To fill the gap of a direct method of observation of toddlers’ receptive and expressive lexical abilities of nouns and predicates for children aged 2–3 in English, the WinG Words in Game test (WinG test; Cattani, Krott et al. 2019) was adapted from PinG, and normed for British English children in the United Kingdom. It provides the monthly norms of four subtasks, including two for comprehension and two for production, of 376 children aged 19–36 months (noun comprehension, predicate comprehension, noun production and predicate production). However, the published normed scores did not include concurrent validity, predictive invariance across genders for the comprehension and production tasks, or clinical cut‐off scores for discriminating children at‐risk with language delay from those with typical development.

The other direct standardised measures of language development available in the United Kingdom either assess older children, are time‐consuming or are restrictive regarding vocabulary assessment. For example, the Preschool Language Scale (PLS‐5, Zimmerman et al. 2014) covers a range of auditory and expressive language through play interaction, including mixed processing skills of sentence items and a handful of vocabulary items. Despite being an excellent instrument for diagnostic purposes, it does not focus on the lexicon, is lengthy and requires extensive training. The other scales examine partial areas of a child's lexicon. The British Ability Scales (BAS3, Elliott and Smith 2011) and the WPPSI (WPPSI‐4, Wechsler 2013) include a vocabulary acquisition index normed only for 30 and 36 months and are limited to assessing knowledge of nouns (not actions, adjectives and adverbs). On the other hand, the British Picture Vocabulary Scale (BPVS III, Dunn et al. 2009), applicable from quite late (36 months), assesses only receptive, but not productive, vocabulary of nouns and verbs. Looking at the indirect observation through parental reports, such as the British adaptations of the MacArthur‐Bates Communicative Development Inventory (MB‐CDI) (Fenson et al. 2007), the norming data of the repertoire of words comprehended and produced for the British population includes infants from 12 to 25 months for the Oxford CDI (Hamilton et al. 2000) or even younger from 8 to 18 months for the UKCDI (Alcock et al. 2020). In this context, there is a clear need for a direct and comprehensive measure of vocabulary knowledge in children aged 2–3 years. Such a tool would provide Early Years Professionals with a reliable and objective means of assessing language development during this critical period. Early identification of language difficulties enables timely intervention, helping children get the best possible start in life (e.g., Law et al. 2017).

The four subtests of the WinG test have demonstrated excellent reliability for internal consistency (with Cronbach alpha between 0.78 and 0.86), for internal consistency via the split‐half method (with Cronbach alpha between 0.62 and 0.77), and internal coherence across the four subtests (Pearson r between 0.64 and 0.78) in the standardisation phase. They were validated against measures of language ability for concurrent validity (Cattani, Krott et al. 2019)^1^. The tool has also been found to be a robust assessment when used online during lockdowns (Nguyen et al. 2025). These findings provide the foundation for the current study, which aimed to establish the full range of psychometric properties of the WinG test.

### 1.2 Gender Differences

Gender differences in vocabulary size were initially reported by Huttenlocher et al. (1991), who examined the role of parental language input in vocabulary acquisition, estimated through audio/video recording of a typical day of several infants between 14 and 26 months. The amount of child‐directed parental input and age predicted the rate of vocabulary growth, with its acceleration being higher in girls than in boys.

Much of the subsequent work on gender differences in the early lexicon is based on parental report measures, with few studies using children's observations in ecologically valid contexts and with direct assessment (see the review from Rinaldi et al. 2021). In the normative data on parental reports of the MB‐CDIs (Fenson et al. 2007), American English‐speaking children reported mean score differences in word comprehension and production tended to be higher for girls than boys by one to two months.

In large cross‐linguistic CDI comparisons of 10 non‐English languages (Eriksson et al. 2012) and expanded work with 22 languages (Frank et al. 2021), girls were reported to produce significantly more words than boys, a consistent effect across almost all languages examined (Frank et al. 2021), which increased with age (Erikson et al. 2012). For word comprehension, however, no statistically significant advantage was found for girls in Erikson et al. (2012), whereas Frank et al. (2021) reported a slight but relatively consistent female advantage. The authors rejected the possibility that these effects were due to parental report bias, acknowledging that comprehension was likely the measure most affected by reporting biases.

Across both studies, boys displayed a greater variability in word comprehension and production than girls, with a tendency of overrepresentation in the lower tails (Chilosi et al. 2023; Wallentin 2020). This is not surprising as boys tend to be more represented than girls in clinical diagnoses of language disorder, stuttering, and autism (Bölte et al. 2023), and outnumber girls by a ratio of 2:1 in the lowest 10th percentile of language production scores at 26 months of age (Wallentin 2020; a similar ratio is reported by Norbury et al. 2016, at age 4).

In sum, girls generally display an earlier emergence of language development and a larger vocabulary than boys, but the size of this gender effect appears to be relatively small. We aim to confirm the commonly observed advantage for girls in word production using the direct assessment of the WinG test and expect only a slight gender difference in word comprehension, if any.

### 1.3 Optimal Ages for Screening

This study presents the adaptation, validity and reliability of the WinG test that measures the comprehension and production of nouns and predicates in 80 items of children aged 19–36 months. The assessment of vocabulary in toddlers is appropriate for screening children who are late talkers, that is, children under the age of 3 who exhibit lexical difficulties and who are potentially at‐risk for language disorder, whilst not yet diagnosed with DLD (Fisher 2017); this conservative approach takes into account the rapid lexical expansion and the great variability among children at that age. Although Law et al. (2000) concluded that the optimal period for accurate screening is 3–5 years, they pointed to the need to screen younger children to help distinguish children with speech and language disorders from late talkers who would catch up in development.

In a systematic review of screening tools for DLD, Sansavini et al. (2021) highlighted a lack of consensus in defining the most appropriate age for the use of diagnostic tools, while emphasising the importance of early developmental surveillance and intervention. However, the review provided indirect indications that the optimal time for screening should be between ages 2 and 3, whereas diagnosis is optimal at around age 4 (Sansavini et al. 2021). Reasons for the difficulty in identifying an appropriate age for a reliable screening are that, firstly, some children with language delays catch up, displaying typical language skills as they grow older, and secondly, children's linguistic output shows wide variability across domains—ranging from phonology to vocabulary and grammar in utterances—at different ages of screening.

### 1.4 Accuracy of Vocabulary Screening Tools: Sensitivity and Specificity

A recent systematic review of the accuracy of language screening tools in preschool children by So and To (2022) found that few tools used direct language assessments administered by trained professionals, whereas in most cases, clinical markers were identified by parents. Among the screening tools that reported concurrent validity, just about 14% achieved good accuracy^2^ in identifying children with and without language disorders; a surprisingly smaller proportion of screening tools (only 2%) validated with children under 4 achieved good accuracy.

Among the commercial language disorder screeners available in North America for children and young people aged 0–21 years (Bao et al. 2024), only 10 reported classification accuracy, with sensitivity ranging from 70%–100% and specificity from 68%–90%. In the United Kingdom, the Early Language Identification Measure (ELIM) was introduced in 2020 by Public Health England as part of the Health Visitors’ 2–2½ year review. It contains a 50‐word list (a parental questionnaire), a section about practitioner's observations and an interview with the parent; tested against the PLS‐4 as the reference standard, its sensitivity reached 0.98 and its specificity 0.63 (Law et al. 2023). The ELIM is clearly a valuable tool for Early Years Professionals, such as Health Visitors and educators, in identifying language difficulties; however, its use is limited to children up to 2.5 years of age and to word production.

In summary, the reviews highlight the scarcity of effective screening tools, a gap further exacerbated by the limited availability of measures with robust psychometric properties. They underscore the importance of early screening for language difficulties, while recognising that a formal diagnosis of DLD is generally more appropriate when the child is between 4 and 5 (Fisher 2017; Sansavini et al. 2021). Further, the current UK standard, the ELIM, relies on parents’ or carers’ subjective assessments of a child's lexical skills and is administered by Health Visitors, who are not trained in language development. While the ELIM is a useful instrument, there is a clear need for a tool administered by professionals with expertise in language development, such as SLTs, to support a multi‐method assessment that integrates the collected information. SLTs may combine parent‐report measures with direct observation of a child's behaviour and responses during structured screening of word comprehension and production up to age 3, thus enabling a more objective and clinically informed assessment.

### 1.5 Aims of the Study

The present study aims to provide new evidence for validating the WinG test, a vocabulary screening tool for 19–36‐month‐old British English‐speaking children (Cattani, Krott et al. 2019). Whereas previous work provided support for the validity and reliability of the four WinG subtests (noun comprehension, predicate comprehension, noun production and predicate production), the current work assesses the psychometric properties and diagnostic accuracy of two aggregate tasks of the WinG test: comprehension (of nouns and predicates) and production (of nouns and predicates). Specifically, the first aim is to confirm the psychometric properties of the dimensions of WinG test comprehension and production by assessing their reliability, internal consistency, concurrent validity and predictive invariance across genders. The second aim is to determine their diagnostic accuracy (i.e., optimal sensitivity and specificity), and to establish clinical cut‐off scores for identifying children with typical vs. at‐risk language development. The final aim of the study is to present developmental trends in percentile scores of toddlers’ aggregate lexical comprehension and production, using 3‐month age bands to increase sample sizes within each group, and examining differences by gender where these emerge.

## 2 Method

### 2.1 Participants and Procedure

Data for the present study were taken from the WinG standardisation (Cattani, Krott et al. 2019) with 406 English‐speaking children aged 19–38 months, selected via opportunity sampling from nurseries, social media and the Babylab facility at the University of Plymouth. Testing was done in nurseries, Babylab and homes in rural (Kent, Essex, Devon and Cornwall), urban and suburban regions (Birmingham, Canterbury, London, Lincoln) of England. Testing was carried out by 13 female British English‐speaking psychology graduates and SLTs who attended a two‐day training prior to the start of data collection. Each child was administered the WinG test, as well as the PLS‐4, for a subset of children randomly selected to match the ages and genders of children who completed the WinG (by the same assessor). All assessors were blind to the WinG output because the project involved norming the WinG test, and scoring was conducted at the end of data collection. The administration procedure was conducted across two sessions of approximately 40 min each, with duration varying according to individual child factors (e.g., fussiness, motivation and fatigue).

Children from the original dataset sample were excluded based on the following conditions: (1) any child exposed to another language than British English from the parents (*N* = 8); (2) being diagnosed with developmental disorders or any health problems which could interfere with language development such as prematurity (less than 36 weeks), low birthweight (less than 2500 g), identifiable speech or identifiable developmental delay (*N* = 5); (3) being older than 36 months (*N* = 11). Moreover, children were excluded from data analyses when they did not complete at least 17 of 20 answers in each WinG subtest or did not cooperate by not responding (including the absence of a gesture production) to five or more consecutive items in the WinG comprehension tasks (*N* = 46).

These exclusion criteria led to removing the data from 17% of the children. The remaining 336 children (*M_age_* = 28.63 months; *SD_age_* = 4.78; 49.7% females) were included in the final sample for the psychometric analyses on the WinG comprehension task. Of them, 313 children (*M_age_* = 28.87 months; *SD_age_* = 4.70; 50.2% females) also completed the production subtests and were included in the analyses on the WinG production task.

Furthermore, a subgroup of 85 children was also screened with the PLS‐4 test (reference standard). Thus, only this subgroup was included in the analyses for diagnostic accuracy. Specifically, 85 participants (*M_age_* = 27.76 months; *SD_age_* = 4.79; 50.6% females) completed both the WinG comprehension noun and predicate subtests and the two PLS‐4 subtests and were therefore included in the analyses for diagnostic accuracy of the WinG Comprehension task. Of them, 81 children (*M_age_* = 28.05 months; *SD_age_* = 4.72; 51.9% females) also completed the WinG production noun and predicate subtasks and were included in the diagnostic accuracy analyses for the WinG production task. Power analysis for the correlation between the new tool and the reference measure indicated that the sample size of 81 is sufficient to detect at least a small to medium effect with 80% power.

About the parental educational background in our sample, 50.5% of parents (55.5% mothers and 45.4% fathers) have a university degree or above; 41.5% (37.6% mothers and 45.7% fathers) have completed high school (A‐level education or equivalent to 13‐grades), and the remaining 8.1% (7.3% mothers and 8.9% fathers) have a general secondary education level or lower (equivalent to 11‐grades or less). National statistics (ONS, 2025) indicate that approximately 43%–48% of UK adults with dependent children hold a degree‐level qualification, with the remaining proportion equally distributed across secondary and lower levels of education, suggesting that our sample's educational distribution broadly reflects current UK demographics.

### 2.2 Instruments

#### 2.2.1 Individual Information

Participants’ age (in months), gender, any known health problems of children and parental education level (1 = secondary school or lower; 2 = completed A‐level; 3 = university degree or higher) were collected. The parental education level was the highest level of education achieved by either mother or father (see Cattani and Celik 2024). Descriptive data are presented in Table 1.

**TABLE 1:** Sociodemographic characteristics of British‐English speaking children by 3‐month age bands.

Participants’ age	19–21	22–24	25–27	28–30	31–33	34–36	Total
	(*n* = 30)	(*n* = 44)	(*n* = 67)	(*n* = 71)	(*n* = 53)	(*n* = 71)	(*n* = 336)
Gender, *n* females *(%)*	12 (40)	22 (50)	35 (52)	35 (49)	29 (55)	34 (48)	167 (50)
*Parental education*							
Secondary education, *n (%)*	0	0	1 (1.5)	8 (11.3)	4 (7.5)	5 (7.0)	18 (5.4)
High school, *n (%)*	8 (26.7)	11 (26.2)	18 (26.9)	22 (31.0)	22 (41.5)	17 (23.9)	98 (29.3)
Degree or higher, *n (%)*	22 (73.3)	29 (69.0)	44 (65.7)	41 (57.7)	27 (50.9)	49 (69.0)	212 (63.5)

*Note*: Parental education level is the highest level of education achieved by either mother or father, not only by mothers or only by fathers: secondary education (mandatory school up to age 16, equivalent to 11‐grades or less); high school education, that is, academic, technical or vocational (up to age 18, equivalent to 13‐grades); degree or higher (over 16 years of education). Data for the educational level of 8 parents are missing. *n* = number of cases; *%* = percentages.

#### 2.2.2 WinG Test

The WinG test (validated for Italian: PinG; Bello et al. 2010, Bello et al. 2012, and for British English children: Cattani, Krott et al. 2019) is composed of four subtests of 22 items each: noun comprehension (NC), noun production (NP), predicate comprehension (PC), and predicate production (PP). The four subtests take roughly 30 min to administer. The first two items of each set, for both comprehension and word production tasks, are used for training, while the remaining 20 items constitute the raw score. The items consist of coloured picture cards depicting an object (e.g., bus) or a context defining an action (e.g., singing), a descriptive word (e.g., short), or a locative word (e.g., behind). For each item, children are requested to indicate the target figure in the comprehension tasks (e.g., “Where is the cat?”, three picture cards are presented) or to name the target image in the production tasks (e.g., “What is she doing?”, one picture card is presented).

After the training, the WinG test starts. Three cards are presented in a random order in a line in front of the child: a comprehension card, a semantic distractor card (corresponding to the production target card), and an unrelated distractor card. The children are first asked to point or touch the named comprehension card; once they point to one of the cards, their result (correct or incorrect) is recorded, with a single attempt permitted. Then, the comprehension and unrelated distractor cards are removed, leaving the production card used for the production task. The assessor asks the child to name the card; a maximum of two naming attempts is permitted. After the child's response or in case of no response, the administrator moves on to the next set of cards. This is repeated for the next set of pre‐test cards, all 20 experimental noun subtest, the two sets of pre‐test cards for the predicate subtest and all 20 experimental predicate cards. Praise is regularly provided, irrespective of the child's answers. The sets of cards are all presented in the fixed order suggested in the WinG manual, and the gradation of difficulty is balanced for the noun and the predicate subtests, such that the first half of each subtest is as difficult as the second half. Not all children remain engaged throughout testing. Following the WinG manual, if a child shows signs of fatigue or gives no response across five consecutive trials, a short break is offered. Testing then resumes from the previous item, with comprehension and production assessed separately; if needed, the session can be completed on another day within one week.

Correct answers were rated as 1 (versus incorrect as 0), so higher raw scores indicate more proficiency in each test. For the comprehension task, the answers were correct if the child pointed, touched, or grabbed the correct picture card. The highest raw score for the collapsed comprehension nouns and predicates subtests is 40 by design. For the production task, the answers were considered “Correct” if the child said the word (singular or plural), the verb (infinitive or conjugated), or the adjective. Also, onomatopoeia (e.g., “*roar*” for “*lion*”) and phonologically simplified forms (e.g., “*brella*” for “*umbrella*”) were scored as correct. Again, the highest raw score for the collapsed production nouns and predicates subtests is 40. For full details on the procedure to administer the tool, see Cattani, Krott et al. (2019).

#### 2.2.3 Preschool Language Scale Fourth Edition

The Preschool Language Scale—UK Edition (PLS‐4; Zimmerman et al. 2009) is a norm‐referenced measure designed to identify language development delays in children from birth to 6 years and 11 months. The PLS‐4 comprises two subscales: Auditory Comprehension (AC; evaluating language comprehension) and Expressive Communication (EC; evaluating language production), each containing sentence processing items and vocabulary items. Child's responses were scored according to the examination manual. The PLS‐4 raw scores for each task were converted into age‐adjusted standard scores to compare the child's abilities with the mean of the population. The PLS‐4 cut‐off criterion to identify children with language disorders corresponds to a standard score of 85 (1 SD below the mean; Zimmerman et al. 2009), which can be applied to each subtest or to the total language score. The psychometric and diagnostic adequacy of this tool has been broadly confirmed in previous research (Zimmerman and Castilleja 2005; Zimmerman et al. 2009), with PLS‐4 total language scores demonstrating good to excellent values of internal consistency (Cronbach's *α* from 0.86 to 0.97), test‐retest stability coefficients (from 0.90 to 0.97), and inter‐rater reliability (99% agreement). The diagnostic accuracy for 48 children aged 3:00–3:11 was a sensitivity of 0.83 and a specificity of 0.88 for the PLS‐4 total language score. However, no sensitivity and specificity values were available for children under 36 months of age (Zimmerman et al. 2009). In the present study, PLS‐4 scores have been used as a reference standard test.

### 2.3 Data Analysis Planning

Data analyses were conducted using SPSS software version 27.0. Preliminarily, the raw scores in each WinG subtest (NC, PC, NP, PP) were converted into age‐adjusted standard scores (mean = 100, SD = 15). The standardisation procedure was conducted separately on 3‐month age bands (i.e., children of 19–21 months; 22–24 months; 25–27 months; 28–30 months; 31–33 months and 34–36 months), thus the raw score of each child was standardised on the mean score of their corresponding age group. Then, the age‐adjusted standard scores were collapsed into two aggregate scores: comprehension (averaging the standard scores in nouns and predicates) and production (averaging the standard scores in nouns and predicates). Item‐level factorial analyses were also run to confirm the statistical adequacy of this decision (see Supplemental materials).

As regards the PLS‐4 AC and EC scales, we converted the raw scores of each scale into age‐adjusted standard scores following the instructions of the PLS‐4 manual. According to clinical cut‐off criteria (PLS‐4 scores of 85; Zimmerman et al. 2009), participants were also classified into two groups: children who scored above the clinical cut‐off in one or both PLS‐4 scales (AC or/and EC ≥ 85) formed the “typical language development” group, while children who scored below the cut‐off in both scales (both AC and EC < 85) formed the “at‐risk language development” group.

Regarding the psychometric properties of the WinG tasks, the reliability in each gender group and in the total sample was estimated with Cronbach's alpha values (benchmarks suggested by Ponterotto and Ruckdeschel 2007). Internal consistency was estimated by average inter‐item correlations (acceptable values > 0.15) and by the minimum item‐total correlation (good discrimination level > 0.20). The concurrent validity of the two WinG tasks was ascertained by their bivariate correlations with the PLS‐4 AC, PLS‐4 EC scales and the PLS‐4 total score. Two moderation regression models were performed to ascertain the predictive gender invariance of the two dimensions. Specifically, Model 1 estimated the predictive gender invariance of the WinG comprehension task. The PLS‐4 AC scale was the criterion variable, gender was entered as a control variable in the first step, and the WinG comprehension task was added as an independent predictor in the second step to ascertain its role in predicting PLS‐4 AC scores. The statistical product between gender (girls = 0, boys = 1) and WinG comprehension was then included in the third step of the regression equation to verify whether the WinG comprehension scores invariantly predicted the PLS‐4 AC scores in boys and girls (predictive gender invariance). Similarly, Model 2 estimated the predictive gender invariance of WinG production, entering the PLS‐EC scale as the criterion variable and the WinG production as the independent predictor.

Regarding gender differences, two univariate analyses of covariance (ANCOVAs) were run to investigate gender differences in the raw scores of comprehension and production tasks, controlling for children's age and parental education. Then, the normative scores in comprehension and production dimensions were computed at the 5^th^, 15^th^, 25^th^, 50^th^, 75^th^ and 95^th^ percentiles in 3‐month age bands, divided for girls and boys if gender differences were found in the ANCOVAs.

As for diagnostic accuracy, a series of sensitivity and specificity analyses were run on the WinG comprehension and production tasks to ascertain the most accurate cut‐off values to identify children with language development delays. Sensitivity represents the probability that the target measure (i.e., the WinG tasks) correctly identifies children with a certain property (e.g., at‐risk language development) when the same property is detected by the reference standard test (true positive cases, TP, divided by the sum of TP and false negative cases, FN). Specificity is the probability that the target measure correctly identifies participants who do not have the property when the property is not detected by the standard (true negative cases, TN, divided by the sum of false positives, FP, and TN cases), that is, does not mistakenly identify typically developing children on the PLS‐4 benchmark test as at‐risk on the WinG tasks.

Two receiver operating curves (ROC) were performed respectively on comprehension and production tasks, entering the PLS‐4 typical (coded as 1) versus at‐risk (coded as 0) groups as reference standards. The adequacy of the area under the curve (AUC; acceptable values > 0.70; Fisher et al. 2003) was determined for both comprehension and production tasks. The ideal cut‐off scores for comprehension and production tasks were then identified at the highest values of Youden *J* index. Values of accuracy (TP + TN / positive + negative) and error rate (FP + FN /positive + negative) were also computed for the eligible cut‐off scores, together with positive predictive values (PPV = TP/TP + FP) and negative predictive values (NPV = TN/FN + TN).

## 3 Results

### 3.1 Reliability and Internal Consistency

Both comprehension and production tasks showed excellent reliability (Cronbach's *α* = 0.87), consistent across genders (see Table 2), with acceptable inter‐item correlations (0.16 in comprehension and 0.25 in production). Item discrimination was adequate for production (0.22) but lower for comprehension (0.13); however, all items contributed positively to overall reliability, supporting the adequacy of the WinG tasks^3^.

**TABLE 2:** Descriptive statistics and reliability values on comprehension and production raw scores.

	*Boys*				*Girls*				*Total sample*			
	*M*	*SD*	*α*	*n*	*M*	*SD*	*α*	*n*	*M*	*SD*	*α*	*N*
1. WinG comprehension	29.29	6.46	0.86	169	31.39	6.31	0.88	167	30.29	6.42	0.87	336
2. WinG production	15.95	7.98	0.86	156	18.44	9.16	0.88	157	17.05	8.69	0.87	313

*Note*: α = Cronbach's alpha values. The benchmarks for reliability (Ponterotto and Ruckdeschel 2007) indicate acceptable α values as > 0.75, and excellent α values as > 0.90, for samples of 100–300 subjects, and scales with 12 or more items.

The high correlation coefficients between the two tasks of noun and predicate in each dimension (comprehension: *r* = 0.57; production: *r* = 0.66) provided support for the decision to aggregate WinG's items in the two dimensions. Item‐level factorial analyses were also run to further confirm the adequacy of this statistical choice (results reported in the Supplemental materials).

### 3.2 Validity and Predictive Invariance Across Gender

Participants who completed the comprehension task and PLS‐4 AC (*n* = 85) were included in the subsequent analyses on concurrent validity and gender invariance of WinG comprehension; those who also completed the production task and PLS‐4 EC (*n* = 81) were included in the production validity analyses. As for the concurrent validity of the WinG test, WinG comprehension and production showed significant positive correlations with PLS‐4 AC, EC, and total scores (all *p* ≤ 0.001), with medium to large effect sizes (see Table 3).

**TABLE 3:** Bivariate correlations of WinG tasks with PLS‐4 scales for concurrent validity

	PLS‐4 AC	PLS‐4 EC	PLS‐4 total
1. WinG comprehension	0.49^*^	0.34^*^	0.44^*^
*n*	85	85	85
2. WinG production	0.44^*^	0.51^*^	0.52^*^
*n*	81	81	81

^*^ *p* ≤ .001. Pearson's *r* coefficients are reported.

Model 1 examined the predictive gender invariance of WinG comprehension task: gender (step 1) was not significant, while WinG comprehension (step 2) explained 25% of the variance in PLS‐4 AC scores and was a significant positive predictor, supporting its validity. The interaction term gender^*^comprehension (step 3) was not significant, indicating predictive gender invariance (see Table 4).

**TABLE 4:** Moderation regression analyses proving predictive gender invariance

	PLS‐4 AC		PLS‐4 EC	
	*ΔR^2^*	*beta*	*ΔR^2^*	*beta*
Step 1	0.001		0.002	
Gender (0 = girl; 1 = boy)		0.03		0.04
Step 2	0.25^*^		0.26^*^	
Gender (0 = girl; 1 = boy)		−0.03		0.04
WinG comprehension		0.50^*^		—
WinG production		—		0.51^*^
Step 3	0.00		0.00	
Gender (0 = girl; 1 = boy)		−0.10		0.06
WinG comprehension		0.51^*^		—
WinG production		—		0.51^*^
Gender*comprehension		−0.07		—
Gender*production		—		0.02
Total *R* ^2^	0.25^*^		0.26^*^	
*N*	85		81	

^*^ *p* < 0.001

Model 2 tested the predictive invariance of the WinG production task: gender (step 1) was nonsignificant, while production (step 2) explained 26.3% of the variance in PLS‐4 EC scores and was a significant positive predictor, supporting its validity. The interaction term gender^*^production (step 3) was not significant, indicating predictive gender invariance (see Table 4).

### 3.3 Gender Differences and Normative Scores

No gender differences were found in parental education (*F*_(1, 327)_ = 0.001, *p* = 0.97). Two ANCOVAs then examined gender differences in comprehension and production raw scores, controlling for age and parental education (as covariates), with sample sizes of 328 and 307, respectively, due to missing data.

For both dimensions, significant covariate effects were found for parental education level (comprehension: *F*_(1, 327)_ = 8.54, *p* = 0.004, *η* ^2^ *_p_* = 0.03; production: *F*_(1, 306)_ = 7.20, *p* = 0.008, *η* ^2^ *_p_* = 0.02) and for children's age (comprehension: *F*_(1, 327)_ = 178.74, *p* < 0.001, *η* ^2^ *_p_* = 0.36; production: *F*_(1,306)_ = 325.34, *p* < 0.001, *η* ^2^ *_p_* = 0.52). Specifically, both comprehension and production scores increased with age and with higher parental education levels. Controlling for these effects, significant gender differences in comprehension and production raw scores were also found (comprehension: *F*_(1, 327)_ = 13.39, *p* < 0.001, *η* ^2^ *_p_* = 0.04; production: *F*_(1, 306)_ = 11.21, *p* = 0.001, *η* ^2^ *_p_* = 0.04), with girls outperforming boys in both tasks. The descriptive statistics by gender are reported in Table 2.

Figures 1a and 1b show WinG comprehension norms by 3‐month age groups and percentiles (at the 5^th^, 15^th^, 25^th^, 50^th^, 75^th^ and 95^th^ percentiles), with boys generally scoring lower than girls. Figures 2a and 2b show a similar pattern for production, particularly at higher percentiles. Overall, comprehension was easier than production, with earlier variability narrowing over time, while production showed steady growth from floor levels with consistent variability.

**FIGURE 1a:**
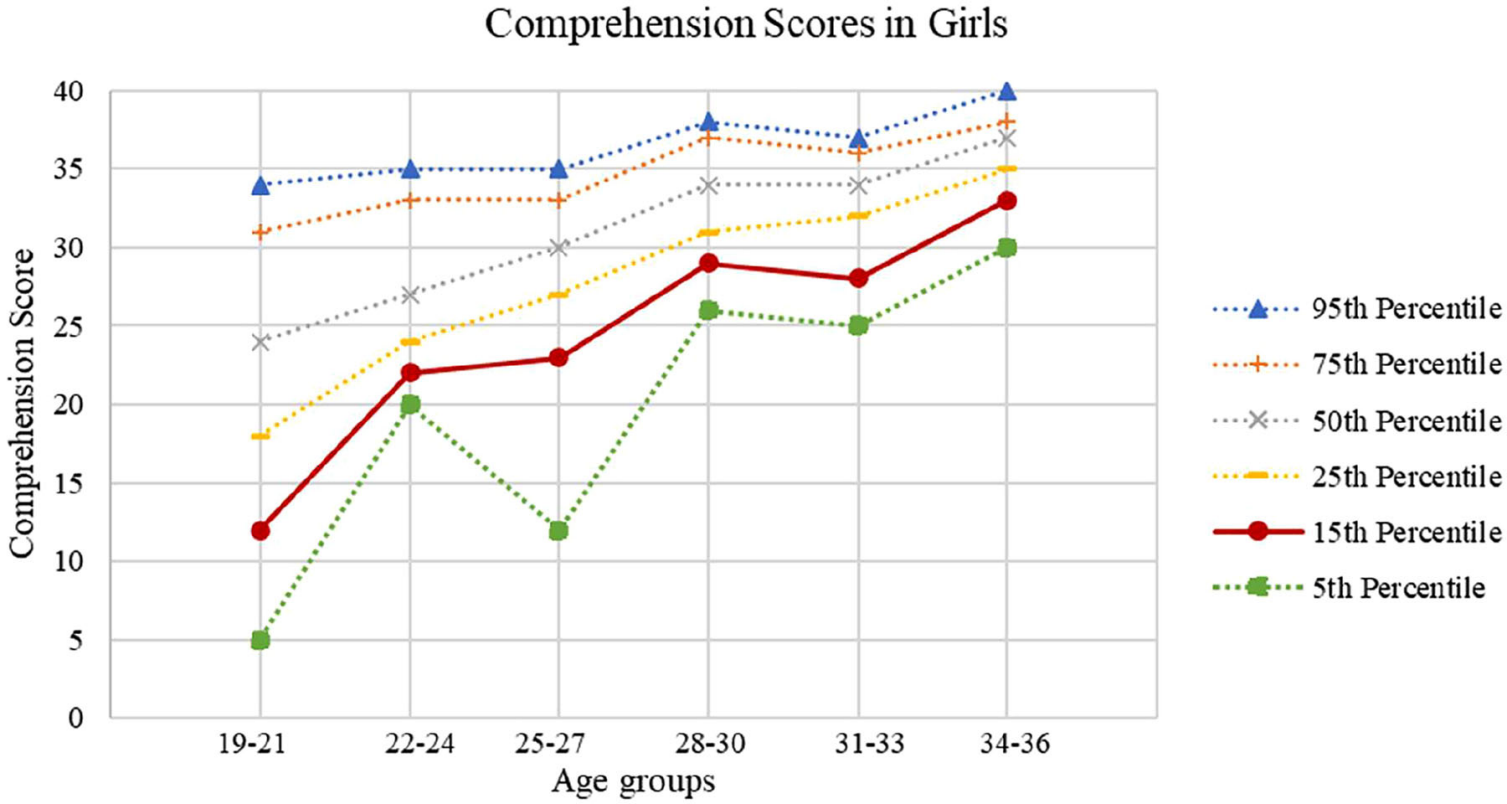
WinG comprehension score norms in girls (*n* = 167).

**FIGURE 1b:**
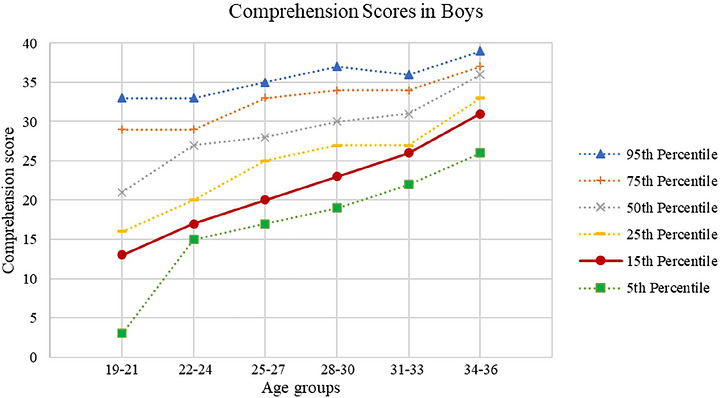
WinG comprehension score norms in boys (*n* = 169).

**FIGURE 2a:**
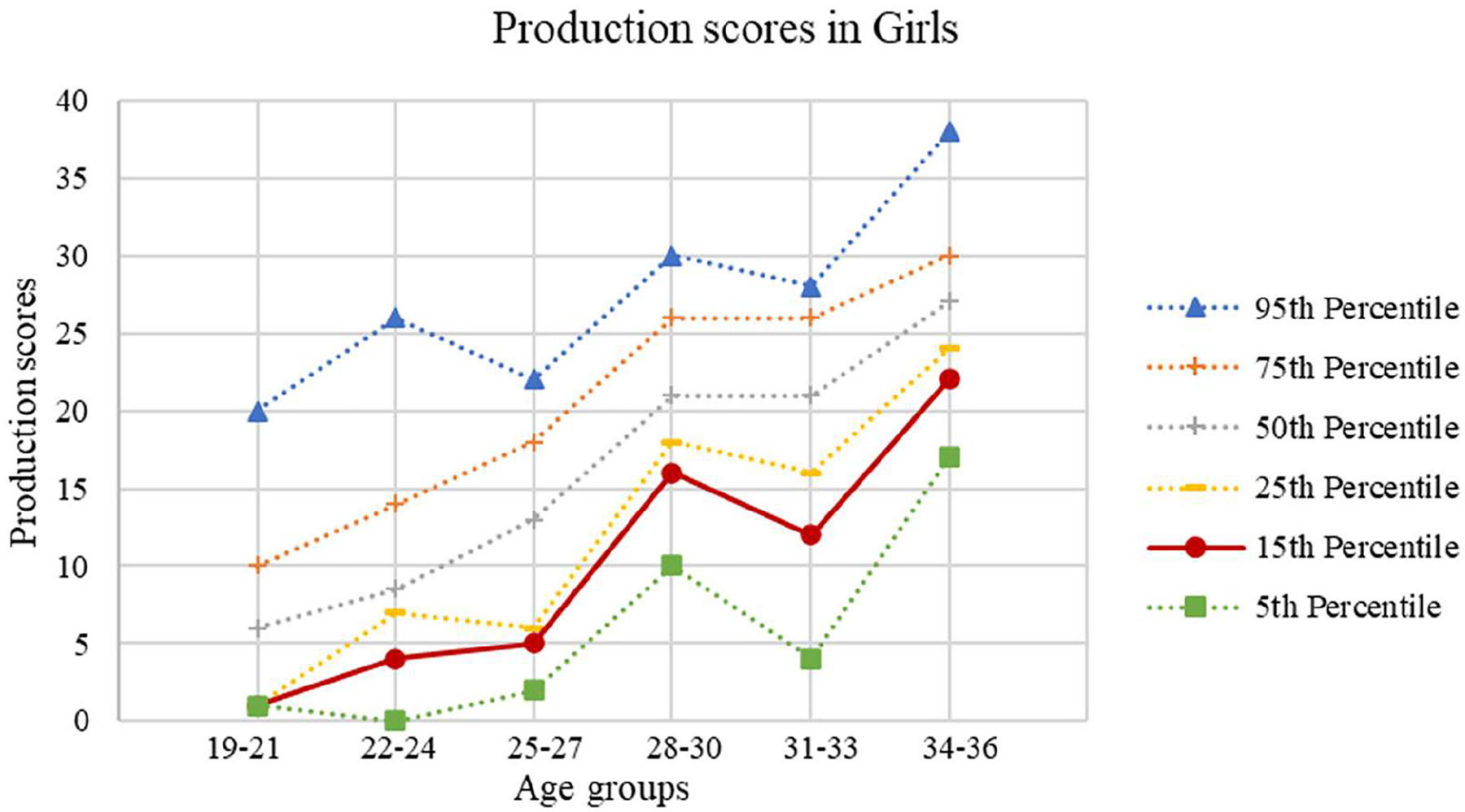
WinG production score norms in girls (*n* = 157).

**FIGURE 2b:**
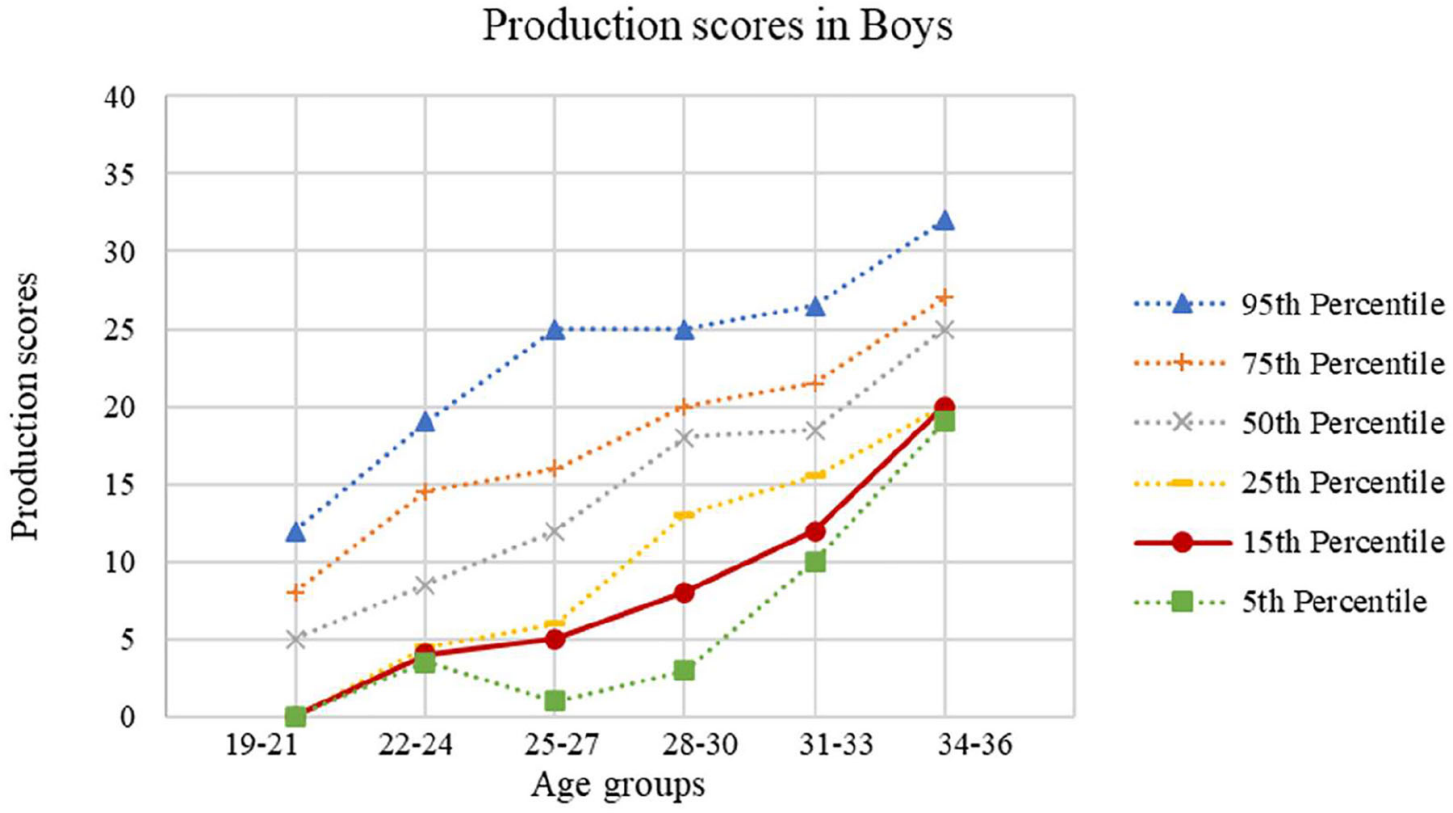
WinG production score norms in boys (*n* = 156).

### 3.4 Sensitivity and Specificity

Participants in the sensitivity and specificity analyses (*n* = 85 comprehension; *n* = 81 production) were classified using the PLS‐4 cut‐off of 85: in comprehension, 76 were typical (89.4%) and 9 at‐risk (10.6%); in production, 72 were typical (88.9%) and 9 at‐risk (11.1%).

ROC analysis for comprehension (*AUC* = 0.76, 95% CI [.564, .952]) showed good discrimination between typical and at‐risk groups, using the PLS‐4 typical vs. at‐risk groups as the reference standard. The optimal balance between sensitivity and specificity cut‐off was identified at the 15th percentile (cut‐off score ≤ 85; Youden *J* = 0.55), yielding sensitivity of 0.88 and specificity of 0.67, with 86% overall accuracy and an error rate of 14%. For example, boys aged 25–27 months scoring ≤ 20 words are classified as at‐risk, and ≥ 21 as typical (Figure 1b; Table SA).

**FIGURE 3:**
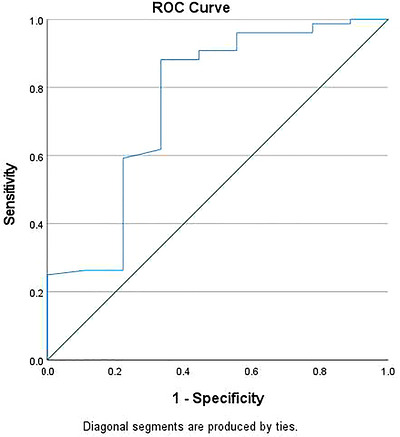
Receiver operating curve (ROC) on the accuracy of WinG comprehension in detecting typical vs. at‐risk language development. *N* = 85 *Note*: The PLS‐4 Typical versus at‐risk groups were entered as reference standard test.

The PPV (positive predictive value) indicates the likelihood that children identified as at‐risk will develop language difficulties, which in this sample was 40%; and the NPV (negative predictive value) reflects the probability that children with negative results remain unaffected, which in this sample was 96%. For low‐prevalence conditions such as developmental language disorders, even highly accurate screening may yield low PPV, emphasising the importance of follow‐up screening (Figure 3; Table 5).

**TABLE 5:** Results of the WinG tasks screening and the PLS‐4 reference standard

	*WinG comprehension*			*WinG production*		
PLS‐4 standard	Typical (> 85)	At‐risk (≤ 85)	Total	Typical (> 85)	At‐risk (≤ 85)	Total
Typical (> 85 in AC and/or EC)	67	9	76	64	8	72
	88.2%	11.8%		88.90%	11.1%	
	TN	FP		TN	FP	
At‐risk (≤ 85 in both AC and EC)	3	6	9	3	6	9
	33.3%	66.7%		33.3%	66.7%	
	FN	TP		FN	TP	
Total	70	15	85	67	14	81

Abbreviations: FN, false negative cases; FP, false positive cases; TN, true negative cases; TP, true positive cases. Typical groups are composed of children scoring over the cut‐off value for each test (scoring > 85 in WinG Comprehension; > 85 in WinG Production; > 85 in one or both PLS‐4 tests).

**FIGURE 4:**
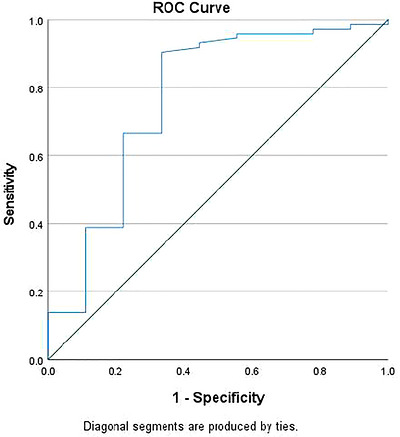
Receiver operating curve (ROC) on the accuracy of WinG production in detecting typical versus at‐risk language development. *N* = 81 *Note*: The PLS‐4 Typical versus at‐risk groups were entered as reference standard test.

ROC analysis for production (*AUC* = 0.77, 95% CI [0.573, 0.964]) showed good diagnostic accuracy. The optimal cut‐off was the 15th percentile (score ≤ 85; Youden *J* = 0.56), yielding sensitivity of 0.89 and specificity of 0.67, with 86% accuracy and an error rate at 13%; PPV was 43% and NPV 96% (Figure 4; Table 5). For example, boys aged 25–27 months scoring ≤ 5 words are classified as at‐risk, and ≥ 6 as typical (Figure 2b; Table SA).

## 4 Discussion

This study examined the psychometric properties and the validity of an adapted screening tool—the *WinG* test—for assessing lexical comprehension and production in 336 British English‐speaking children aged 19–36 months, grouped into 3‐month age bands. This tool targets word‐level tasks involving nouns, adjectives, adverbs and verbs. The study also examined gender differences in performance. The development of the tool was driven by the critical role of lexical development in children's early language skills. Although it is designed for use by healthcare professionals, early years educators, psychologists and researchers to directly observe and assess a child's vocabulary in comparison to age‐matched peers, it is especially well‐suited for speech and language therapists (SLTs) who require a fully developed tool to screen for lexical difficulties through a direct assessment.

### 4.1 Reliability and Internal Consistency

The validation results demonstrate that the tool provides a reliable measure of children's lexical comprehension and production. The individual items in the comprehension and production tasks target lexical categories that typically emerge in the vocabularies of children between 2 and 3 years of age. The WinG test shows good internal consistency (both tasks at Cronbach's α = 0.87). The reliability of a test is a primary driver of its specificity and sensitivity (Edward et al. 2022; Hulme et al. 2024). In this regard, the WinG test demonstrates strong reliability.

### 4.2 Gender Differences and Normative Scores

We found small but significant gender differences, with girls outperforming boys in comprehension and production. These results, based on direct observation by trained assessors, align with parental reports of vocabulary differences across 22 languages (Frank et al. 2021). Several authors report a clinical overrepresentation of boys in the lower tails of language skills (Chilosi et al. 2023; Wallentin 2020). However, our findings revealed that boys generally performed worse across different ages and percentile ranges.

Girls showed high variability in comprehension at 25–27 months due to a few low‐performing cases. In production, uneven development between 25 and 33 months was observed in both sexes, suggesting greater uncertainty at the lower tails during this period, while higher‐level performance stabilised from 25 months.

In the comprehension task (Figures 1a and 1b), boys consistently performed below girls across most percentiles. In the production task (Figures 2a and 2b), boys also lagged behind, with differences most pronounced at the higher percentiles.

### 4.3 Validity and Predictive Invariance Across Gender

We asked whether the WinG comprehension and production tasks have concurrent validity for the 19–36‐month‐olds. This was assessed by pseudo‐randomly selecting a sub‐sample of children to complete an additional task, the PLS‐4. The concurrent validity of the WinG comprehension and production tasks was strong, showing significant positive correlations with the reference standards—auditory, expressive, and PLS‐4 total scores (i.e., the sum of auditory and expressive items)—with moderate‐to‐large effect for comprehension and large effect sizes for production. Two regression models substantiated the validity of the WinG comprehension and production tasks, establishing their predictive invariance by gender. The scores of the WinG comprehension task explained a significant 25% of the variance in the PLS‐4 auditory comprehension, while the scores of the WinG production task explained 26.3% of the variance in PLS‐4 expressive scores. Gender, entered as a moderator in each regression model, confirmed that WinG test scores (comprehension and production) were valid and consistent with PLS‐4 subtests for both boys and girls.

In summary, this tool provides a valuable starting point for identifying difficulties in early receptive and expressive lexical skills through direct assessment by an SLT. While parental report tools—such as the MB‐CDIs and their numerous adaptations in spoken and signed languages—have proven valuable for characterising early gestural, receptive, and expressive vocabulary development from the earliest stages of life, they primarily rely on parents’ subjective (though important) judgments based on close, daily contact with their child. A validated parent report remains a cost‐effective and valuable tool for screening those children who are unwilling to cooperate during direct assessment testing; however established parent‐report instruments for the British population are available only for children up to the age of 25 months (Oxford CDI, Hamilton et al. 2000) and up to 29 months for production only (ELIM, Law et al. 2023), hence are unavailable for the whole span of the third year, a key age for monitoring lexical development.

### 4.4 Sensitivity and Specificity

The optimal cut‐off was determined as the point that maximised the Youden *J* index, which balances sensitivity and specificity. Hence, the chosen cut‐off was set at the 15^th^ percentile for both the WinG comprehension and production tasks, with a fair sensitivity for the comprehension task (0.88) and good sensitivity for the production task (0.89), but below fair specificity for the comprehension and production tasks (both at 0.67) (note that specificity is higher than the 0.63 reported for the ELIM tool, Law et al. 2023; and no data is available for children under 36 months for the PLS‐4, Zimmerman et al. 2009). Hence, the tool maximises sensitivity at the expense of specificity (as is also the case in the ELIM where sensitivity is at 0.98). That is, most children with difficulties in language comprehension and production, as identified through the PLS‐4, were accurately identified using the WinG test. However, this came at the cost of a higher false‐positive rate, meaning that some children were flagged for further observation even though they did not have true language difficulties.

The recent review by So and To (2022) reported poor values on the sensitivity and specificity of language tests, particularly for the assessment of early vocabulary, with sensitivity ranging from 50%–94%, and specificity from 45%–96%. Indeed, consistent with the review, our work reported low specificity and acceptable sensitivity, foregrounding the difficulty of achieving high accuracy in language screening tools for children under four years old. The rapid expansion of linguistic abilities in the second and third years of life, along with ample variability among children, could explain the low accuracy values. This argument may not be entirely satisfying, because the review by Bao et al. (2024) with screeners for children to adults aged 0–21 reported fair sensitivity and specificity, suggesting that the criteria based on Plance and Vance for concurrent validity (1994) may be too selective for children and need to be revised and adjusted for screening of language abilities. Recently, other authors deliberately decided not to report sensitivity and specificity evidence in the validation of the Language Screen as a diagnostic test for DLD because they considered language disorders to be dimensional rather than separated by a cut‐off index (Hulme et al. 2024; Snowling and Hulme 2024). As Snowling and Hulme (2024) argued, placing a cut‐off is to some extent arbitrary, as DLD might be best thought of as a continuum, rather than a distinct category, with no clear cut‐off points. Indeed, the absence of a choice for a universally accepted cut‐off (e.g., −1.00 SD; −1.25 SD and −1.50 SD) in language screening tests stems from the difficulty researchers and clinicians face and hence has significantly influenced the accuracy values for a consistent procedure for selecting children with language delay. In most cases, the standard reference was represented by experts’ clinical judgment.

We reported the sensitivity and specificity of the WinG tool as preliminary indicators of language impairment risk and to guide decisions about further evaluation. Given its high sensitivity, the priority was to ensure that children identified as not at‐risk truly had no language difficulties, as they were unlikely to receive follow‐up. In practice, slightly lower specificity can be tolerated (Bao et al. 2024), although this increases false positives and may lead to unnecessary evaluations and family concern.

Importantly, not all late talkers develop persistent language disorders (Rinaldi et al. 2021). While some children catch up, others, if not identified early, may face later difficulties, including in reading, with long‐term impacts on academic, mental health, and employment outcomes (Leonard 2014). Hence, the long‐term costs of early developmental difficulties—later reflected in reading, school behaviour, and employment outcomes—could be substantially reduced through early identification and intervention to support language development (Bello et al. 2012; Mattson et al. 2001).

### 4.5 Limitations

This work has some limitations. First, the broad age range (19–36 months) and variability in early language development, combined with a sample of 336 children, may limit the strength and generalisability of the diagnostic accuracy findings. However, 3‐month age bands ensured adequate group sizes, especially from 25 months onwards, where scores stabilised, and allowed gender‐stratified norms. We also did not examine parental education effects, as the sample was skewed towards higher levels. Larger, more diverse samples would allow finer stratification and more detailed analyses of sensitivity and specificity, particularly by including families from less advantaged socio‐economic backgrounds.

Second, a small number of children were unable to complete the assessment due to limited cooperation during the production tasks, particularly among the youngest children aged 19–24 months, as also evidenced by floor‐level scores. Such exclusions are common in direct assessment studies with very young children; this should be considered when assessing the task's feasibility in routine clinical contexts. We therefore recommend administering the production task to children over 24 months of age. In contrast, the comprehension task appears suitable across the full age range, although children over 34 months tend to cluster in the upper percentiles, often reaching ceiling scores on the WinG test.

Third, the diagnostic validation of the WinG test relies on a subsample of approximately 85 children. Given the age range of knowledge expansion for receptive and expressive vocabulary development and the variability in early language development, we acknowledge that a larger sample would yield narrower confidence intervals for estimates such as sensitivity and specificity, and we interpret these indices with appropriate caution. However, this sample size is within the range commonly reported in validation studies of early language measures and provides adequate power to detect moderate to large correlations with the reference standard tool. Relatedly, the PLS‐4 was selected for concurrent validity, despite being a broad language assessment rather than a specific reference standard for lexical skills. The choice of the PLS‐4 was constrained by the lack of available comparisons with established parent‐report instruments in the UK; for example, the use of CDIs as clinical tools is not reliably established, according to a recent review by Eriksson (2023). Therefore, the added value of a directly administered tool like the WinG test remains to be determined through longitudinal follow‐up studies to examine the predictive validity of WinG scores for later language outcomes, in readiness for the diagnosis of DLD.

Finally, in the coding process, the phonologically altered forms of the target words or simplified forms with similar yet intelligible phonology (e.g., ‘*brella*’ instead of ‘*umbrella*’) were scored as correct. Approximate productions are common at a stage where children are in the process of acquiring the concepts underlying target words. Cattani and Bello (2026) observed that children sometimes produce phonologically deviant forms—unintelligible words—that may reflect underlying semantic knowledge, whether accurate or not, but are not recognised by adults as valid target words. Between 24 and 30 months, improvements in the phonological repertoire are evidenced by a decline in unintelligible responses and an increase in more accurate, intelligible productions, including phonologically altered or simplified forms that retain recognisable phonological patterns.

### 4.6 Clinical Implications

For SLTs, the WinG screening tool offers several practical advantages. First, it provides sex‐specific norms for girls and boys, reducing the risk of overdiagnosing boys as being at‐risk of language delays and underdiagnosing girls. Second, including both comprehension and production tasks allows clinicians to differentiate between representational lexical knowledge and expressive skills, which may support more nuanced clinical decision‐making. Children who score low on both comprehension and production are at elevated risk of a broad, persistent language disorder and should be prioritised for comprehensive assessment and early intervention. Children with stronger comprehension than production typically show an expressive delay and often have a more favourable prognosis, whereas children with weaker comprehension than production are at higher risk of persistent language difficulties and warrant closer assessment and monitoring (e.g., Bishop 2014). In the concurrent validity data, the WinG tool and the PLS‐4 identified six at‐risk children (3 girls and 3 boys) in comprehension and production; among them, five were identified outside the cut‐off both in comprehension and production tasks.

Third, early screening as allowed by WinG test prompts early intervention for optimal effectiveness, as toddlers with language delay can show substantial improvement following early intervention (Vermeij et al. 2023). Children younger than 3 years of age at the start of an intervention stabilise or even improve language proficiency on receptive syntax, receptive vocabulary, expressive syntax and expressive vocabulary during the intervention, indicating that the language gap between these children and typically developing children does not widen further (Vermeij et al. 2023).

Fourth, the aggregation of noun and predicate items (verbs, adjectives and adverbs) within each modality provides a clinically meaningful index of lexical–semantic organisation rather than isolated word knowledge. Given the developmental shift from object‐based to relational vocabulary during this age range, performance across lexical categories may help identify children whose semantic organisation is less advanced than expected for their age band.

Finally, the provision of age‐banded norms and empirically derived cut‐off scores allows the WinG test to function as a structured screening instrument within early identification pathways. The screening tool may be particularly useful in settings where direct, standardised assessment by trained professionals is preferred over reliance solely on clinical judgement, given the lack of validated parental reports for the third year, as is the case in the United Kingdom. Importantly, the WinG test is not intended to replace a comprehensive diagnostic assessment but to support a screening for early decision‐making regarding monitoring, referral and intervention planning.

In conclusion, we have presented data from a large sample of British‐English speaking children, assessed with the WinG test to screen their comprehension and production of words, providing SLTs and researchers with a useful, standardised tool. Whilst the comprehension task is accessible to children of all ages, the production task is suitable for children aged 25 months or older. It offers the SLTs a reliable tool for early screening of toddlers’ lexical skills, supporting the identification of language delays towards a formal diagnosis of DLD, monitoring progress following intervention, and assessing school readiness by age 4.

## Ethics Statement

Ethical approval for this study was obtained from the Faculty of Health, Psychology, University of Plymouth (UK) Ethics Committee (APPROVAL NUMBER 14/15‐312) and by the Department of Developmental and Social Psychology, Sapienza University of Rome (Italy) Ethics Committee Prot. n. 0001606 del 12/12/2022 ‐ [UOR: SI000042 ‐ Classif. VII/15]. All children and their parents were informed in detail about the aims of the study, the voluntary nature of their participation, and the right to withdraw from the study at any time. The children's parents gave informed written consent for participation in the study, data analysis, and data publication. The children gave informal verbal consent to participate; if the child, for any reason, did not want to continue, the study was stopped.

## Conflicts of Interest

The authors declare no potential conflicts of interest with respect to the research, authorship, or publication of this article.

## Supporting information




**Supplementry Table A**. Comprehension and production raw scores of girls and boys at 3‐month age bands at 15th percentile.

## Data Availability

The datasets generated during and/or analysed during the current study are available from the corresponding author on reasonable request.

## Endnotes

1 For the concurrent validity, positive and moderate high and relations with a high level of significance (*p* < .01) were obtained between the two comprehension subtests and the BPVS III (Dunn et al. 2009, Pearson *r* between .37 and .53) and with the two production subtests with the SETK‐2 Sprakentwicklungstest‐2 (Grimm, 2000; Pearson *r* = .80). These data cannot be used here as the BPVS III was outside the age range for almost all children. The German‐adapted SETK‐2, initially piloted at the universities of Manchester and Leipzig, showed equivalent performance in the raw scores for 30‐month‐old German and English children, but it has no norms.

2 Based on Plante and Vance (1994), Adequate or Fair = over 0.8 in both sensitivity and specificity, and Good = over 0.9 in both sensitivity and specificity.

3 Reliability and internal consistency indexes were further computed in the six age subgroups. Acceptable, good and excellent reliability values were found across age groups (Cronbach's α ranging from .73 to .90 in comprehension and from .67 to .80 in production), while the internal consistency showed more variability across age bands (values of average inter‐item correlations from .08 to .26; values of the minimum item‐total correlations from .01 to .16). These lower values might be due to the small number of children in each age group, which made the correlations unstable. This can be considered a limitation of the present study.
