# Supplementary material for: An Early Lexical Screening Tool for British English: Psychometric Properties and Clinical Utility
Source: Int J Lang Commun Disord. 2026 Jul 7;61(4):e70287. doi: 10.1111/1460-6984.70287 (PMC13339757; doi:10.1111/1460-6984.70287)
Supplement: Supplementary file 1 — Supplementry Table A. Comprehension and production raw scores of girls and boys at 3‐month age bands at 15th percentile. [file JLCD-61-0-s001.docx]

**Supplemental materials**

**Item-level factorial analyses on WinG’s tasks**

*1.1 Exploratory factor analyses*

Two principal component analyses (PCA) were performed on the WinG items, respectively for the two aggregate dimensions of comprehension (nouns and predicates) and production (nouns and predicates), in order to ascertain their unidimensionality. The Kaiser-Meyer-Olkin (KMO) indexes and the Bartlett sphericity tests preliminarily suggested the adequacy of the items for factor analyses both in comprehension (KMO = .85, Bartlett *p* < .001) and in production (KMO = .92, Bartlett *p* < .001). The first PCA, conducted on the comprehension items, indicated the presence of one single factor explaining the 18.8% of variance (Eigenvalue > 1). The second PCA, conducted on the production dimension, also revealed the presence of one single factor, explaining the 26.3% of variance.

*1.2 Confirmative factor analysis*

A CFA was also run on the 80 items of the WinG test (i.e. 40 items for comprehension and 40 items for production), to provide support for the presentation of a two-factor structure. Data analyses were conducted using the Mplus software version 8.0. Considering the dichotomous nature of WinG items (0 = fail; 1 = correct), a diagonally weighted least squares estimator (WLSMV) for categorical data was applied. Since the chi-square (*χ^2^*) statistic is sensitive to sample size, we also considered the following indicators to estimate the model fit: the relative chi-square index (χ^2^/df ; acceptable values < 5; Hair et al., 1998), the comparative fit index (CFI) and the Tucker-Lewis Index (TLI), which values > 0.90 suggest an acceptable model fit (Hu & Bentler, 1999), and the root mean square error of approximation (RMSEA), which should be < 0.08 for a good fit (Kaplan, 2000). Models’ comparison was conducted using the CFI difference test (ΔCFI), which values ≥ .01 are considered significant.

The CFA models were verified on the subsample of 313 children (50.2% girls) which completed all tasks both on comprehension and on production WinG’s items. The first CFA model investigated the adequacy of a bifactor model (Model 1) including two correlated dimensions (i.e. comprehension and production). The model’s fit turned out to be a good description of our data, with the following values: χ^2^(3079) = 3232.354; χ^2^/df = 1.05; CFI = .984; TLI = .983; RMSEA = .013. A second model was also estimated (Model 2), in which the two dimensions were not correlated. Model 2 obtained poor fit indexes: χ^2^(3080) = 7342.693; χ^2^/df = 2.38; CFI = .543; TLI = .531; RMSEA = .066; and it was also significantly worse than Model 1, ΔCFI = .44. Thus, Model 1 was accepted as the most adequate description of our data.

Model 1 was also tested for structural invariance across gender groups, and specifically configural, scalar and strict gender invariance were investigated, imposing increasing equality constraints to model parameters. Configural gender invariance was confirmed by adequate fit indexes, χ^2^(6158) = 6354.838; χ^2^/df = 1.03; CFI = .972; TLI = .972; RMSEA = .014. Scalar invariance was also supported, χ^2^(6234) = 6449.431; χ^2^/df = 1.03; CFI = .970; TLI = .969; RMSEA = .015; ΔCFI _M_ *_configural_* _- M_ *_scalar_* = .002. Strict factorial invariance was confirmed too, χ^2^(6274) = 6494.821; χ^2^/df = 1.03; CFI = .969; TLI = .969; RMSEA = .015; ΔCFI _M_ *_scalar_* _- M_ *_strict_* = .001.

**Table A**. Comprehension and production raw scores of girls and boys at 3-month age bands at 15th percentile.

|  |  | **Comprehension** | |  | **Production** | |
| --- | --- | --- | --- | --- | --- | --- |
|  |  | *Girls (n = 167)* | *Boys (n = 169)* |  | *Girls (n = 157)* | *Boys (n = 156)* |
| *Age groups* |  |  |  |  |  |  |
| 19-21 |  | 12 | 13 |  | 1 | 0 |
| 22-24 |  | 22 | 17 |  | 4 | 4 |
| 25-27 |  | 23 | 20 |  | 5 | 5 |
| 28-30 |  | 29 | 23 |  | 16 | 8 |
| 31-33 |  | 28 | 26 |  | 12 | 12 |
| 34-36 |  | 33 | 31 |  | 22 | 20 |
